# Cohort profile: the BangladEsh Longitudinal Investigation of Emerging Vascular and nonvascular Events (BELIEVE) cohort study

**DOI:** 10.1136/bmjopen-2024-088338

**Published:** 2025-01-22

**Authors:** Rajiv Chowdhury, Nusrat Khan, Lisa Pennells, Maria L C Iurilli, Md Taslim Uddin Miah, Md Mostafa Monower, Khan Mohammad Thouhidur Rahman, Sharraf Samin, Kazi Nazmus Saqeeb, Ishrat Tasmin, Eleanor Farrow, Samantha Farrow, Ank Michielsen, Catherine Perry, Sarah Spackman, Charlotte van Coeverden, Matthew Walker, Tahmeed Ahmed, James Ajioka, Khondker Abdul Abdul Awal, Adam S Butterworth, Evangelia Chatzidiakou, Jörg Feldmann, Richard Fenner, Meerjady Sabrina Flora, Tuhin Haque, Sarah Hawkes, Syed Shariful Islam, Sirajul Islam, Roderic L Jones, Stephen Kaptoge, Kamrul Hasan Khan, Lawrence King, Shammi Luhar, Abdul Malik, Fazila-Tun-Nesa Malik, Ruchira T Naved, Aliya Naheed, Olalekan Popoola, Rubhana Raqib, Tahmina Shirin, Stephen Sutton, Kim Robin van Daalen, Angela Wood, Simon Griffin, Nicholas Mascie Taylor, Md Khalequzzaman, Md Alfazal Khan, Sohel Reza Choudhury, Emanuele Di Angelantonio, John Danesh, BELIEVE Study, Israt Akter

**Affiliations:** 1 Stempel College of Public Health and Social Work, Florida International University, Miami, Florida, USA; 2 Population Health Sciences Institute, Newcastle University Population Health Sciences Institute, Newcastle upon Tyne, UK; 3 British Heart Foundation Cardiovascular Epidemiology Unit, Department of Public Health and Primary Care, University of Cambridge, Cambridge, UK; 4 Victor Phillip Dahdaleh Heart and Lung Research Institute, University of Cambridge, Cambridge, UK; 5 Directorate General of Health Services, Cumilla Medical College, Cumilla, Bangladesh; 6 Deapartment of Epidemiology & Research, National Heart Foundation Hospital and Research Institute, Dhaka, Bangladesh; 7 Department of Public Health and Informatics, Bangabandhu Sheikh Mujib Medical University, Dhaka, Bangladesh; 8 Department of Health Promotion, Education, and Behavior, Arnold School of Public Health, University of South Carolina, Columbia, South Carolina, USA; 9 Nutrition Research Division, International Centre for Diarrhoeal Disease Research Bangladesh (icddr,b), Dhaka, Bangladesh; 10 Department of Public Health and Primary Care, University of Cambridge, Cambridge, UK; 11 Office of the Executive Director, International Centre for Diarrhoeal Disease Research Bangladesh (icddr,b), Dhaka, Bangladesh; 12 Department of Pathology, University of Cambridge, Cambridge, UK; 13 National Heart Foundation Hospital and Research Institute, Dhaka, Bangladesh; 14 British Heart Foundation Centre of Research Excellence, University of Cambridge, Cambridge, UK; 15 National Institute for Health and Care Research Blood and Transplant Research Unit in Donor Health and Behaviour, University of Cambridge, Cambridge, UK; 16 Health Data Research UK Cambridge, Wellcome Genome Campus and University of Cambridge, Cambridge, UK; 17 Yusuf Hamied Department of Chemistry, University of Cambridge, Cambridge, UK; 18 TESLA-Analytical Chemistry, Institute of Chemistry, University of Graz, Graz, Austria; 19 Centre for Sustainable Development, Department of Engineering, University of Cambridge, Cambridge, UK; 20 National Institute of Preventive and Social Medicine, Dhaka, Bangladesh; 21 Institute for Global Health, University College London, London, UK; 22 Health Systems and Population Studies Division, International Centre for Diarrhoeal Disease Research Bangladesh (icddr,b), Dhaka, Bangladesh; 23 Department of Economics, University of Massachusetts Amherst, Amherst, Massachusetts, USA; 24 Maternal and Child Health Division, International Centre for Diarrhoeal Disease Research Bangladesh (icddr,b), Dhaka, Bangladesh; 25 Non Communicable Diseases, Nutrition Research Division, International Centre for Diarrhoeal Disease Research Bangladesh (icddr,b), Dhaka, Bangladesh; 26 Institute of Epidemiology, Disease Control and Research (IEDCR), Dhaka, Bangladesh; 27 Behavioural Science Group, Department of Public Health and Primary Care, University of Cambridge, Cambridge, Cambridgeshire, UK; 28 Global Health Resilience Group, Barcelona Supercomputing Center, Barcelona, Spain; 29 Cambridge Centre of Artificial Intelligence in Medicine, University of Cambridge, Cambridge, UK; 30 MRC Epidemiology Unit, University of Cambridge, Cambridge, UK; 31 Health Data Science Centre, Human Technopole, Milan, Italy; 32 Department of Human Genetics, Wellcome Sanger Institute, Hinxton, UK

**Keywords:** EPIDEMIOLOGIC STUDIES, Observational Study, Chronic Disease

## Abstract

**Abstract:**

**Purpose:**

Bangladesh has experienced a rapid epidemiological transition from communicable to non-communicable diseases (NCDs) in recent decades. There is, however, limited evidence about multidimensional determinants of NCDs in this population. The BangladEsh Longitudinal Investigation of Emerging Vascular and nonvascular Events (BELIEVE) study is a household-based prospective cohort study established to investigate biological, behavioural, environmental and broader determinants of NCDs.

**Participants:**

Between January 2016 and March 2020, 73 883 participants (aged 11 years or older) were recruited from 30 817 households across urban, urban-poor (‘slum’) and rural settings in Bangladesh. A structured questionnaire was administered by trained personnel recording participants’ demographic, socioeconomic, behavioural, medical, environmental and other factors. Anthropometric measurements and blood pressure were recorded for each participant. Biological specimens were collected and aliquoted for long-term storage and analysis.

**Findings to date:**

Of the 73 883 study participants (mean [SD] baseline age: 39 [15] years), 43 470 (59%) were females, and 38 848 (52%) had no or only primary-level education. Focusing only on the 65 822 adult participants aged 20–79 years at baseline, 15 411 (23%) reported being diagnosed with hypertension; 10 578 (16%) with type 2 diabetes and 7624 (12%) with hypercholesterolaemia. Age and sex-standardised prevalences of these conditions were much higher in urban than slum and rural settings. Overall, the mean (SD) body mass index (BMI) was 25 (5) kg/m^2^, with 10 442 (16%) participants aged 20–79, classified as obese (ie, BMI≥30 kg/m^2^). Mean BMI was also higher in urban than slum and rural areas.

**Future plans:**

The collection of information during the baseline visit was completed in 2020. Regular longitudinal follow-up is ongoing for ascertainment and adjudication of a range of fatal and non-fatal health outcomes among participants. This cohort will provide a powerful resource to investigate multidimensional determinants of incident NCDs across diverse settings in Bangladesh, helping to advance scientific discovery and public health action in an archetypal low-middle-income country with pressing public health needs.

**STRENGTHS AND LIMITATIONS OF THIS STUDY**

- Largest bioresource for non-communicable diseases (NCDs) and related traits in Bangladesh, configured to investigate multidimensional determinants of NCDs across urban, slum and rural settings.
- Comprehensive recording of questionnaire-based data, including demographic, socioeconomic, behavioural, medical, environmental and other factors, as well as physical measurements.
- Collection of a range of biological samples—including serum, plasma, whole blood and nail samples—stored in long-term repositories.
- Use of stored biological samples to enable the study of many genomic and molecular factors together with other information, laying the foundations to advance scientific discovery and public health action.
- The study is not necessarily representative of the general Bangladesh population and could be limited by misclassification of disease outcomes and by loss of contact with some participants during follow-up.

## Introduction

 Non-communicable diseases (NCDs)—including cardiovascular diseases, diabetes, cancer and chronic respiratory diseases—are the leading causes of premature death in low-income and middle-income countries (LMICs).^1 2^ In particular, South Asia has recorded a higher number of life-years lost due to premature NCDs than any other global region, a situation which reflects both the region’s large population and the relatively young age at which NCD deaths tend to occur in this region.^1 3^ Furthermore, while NCD mortality rates have decreased during recent decades in most countries, they remain at a high level in the South East Asia region.^4 5^ Better understanding of the multidimensional determinants of NCDs in South Asia should, therefore, inform the development of appropriately tailored strategies for disease prevention and control.

There is, however, limited evidence available on NCD determinants for many South Asian populations.^2 6^ For example, Bangladesh, a country with 170 million people, is one of the least studied most densely-populated countries with regard to NCDs, despite steep and sustained increases in NCDs risk factors and incidence during recent decades.^7 8^ In 2021, 74% of all adult deaths in Bangladesh were attributed to NCDs.^7 9 10^ The high burden of NCDs in Bangladesh is not just of local public health concern, as mortality due to NCDs has been reported to be more than two times higher among Bangladeshis living in western regions compared with host populations.^11^ An important challenge is, therefore, to establish informative epidemiological resources in a rigorous manner to evaluate risk factors among Bangladeshis.

The present report provides a description of the methods used in the establishment of the BangladEsh Longitudinal Investigation of Emerging Vascular and nonvascular Events (BELIEVE) study. It also describes the baseline characteristics of the study population recruited so far and outlines the rationale for the study’s further development.

### Cohort description

#### Study design and participants

**Figure 1:**
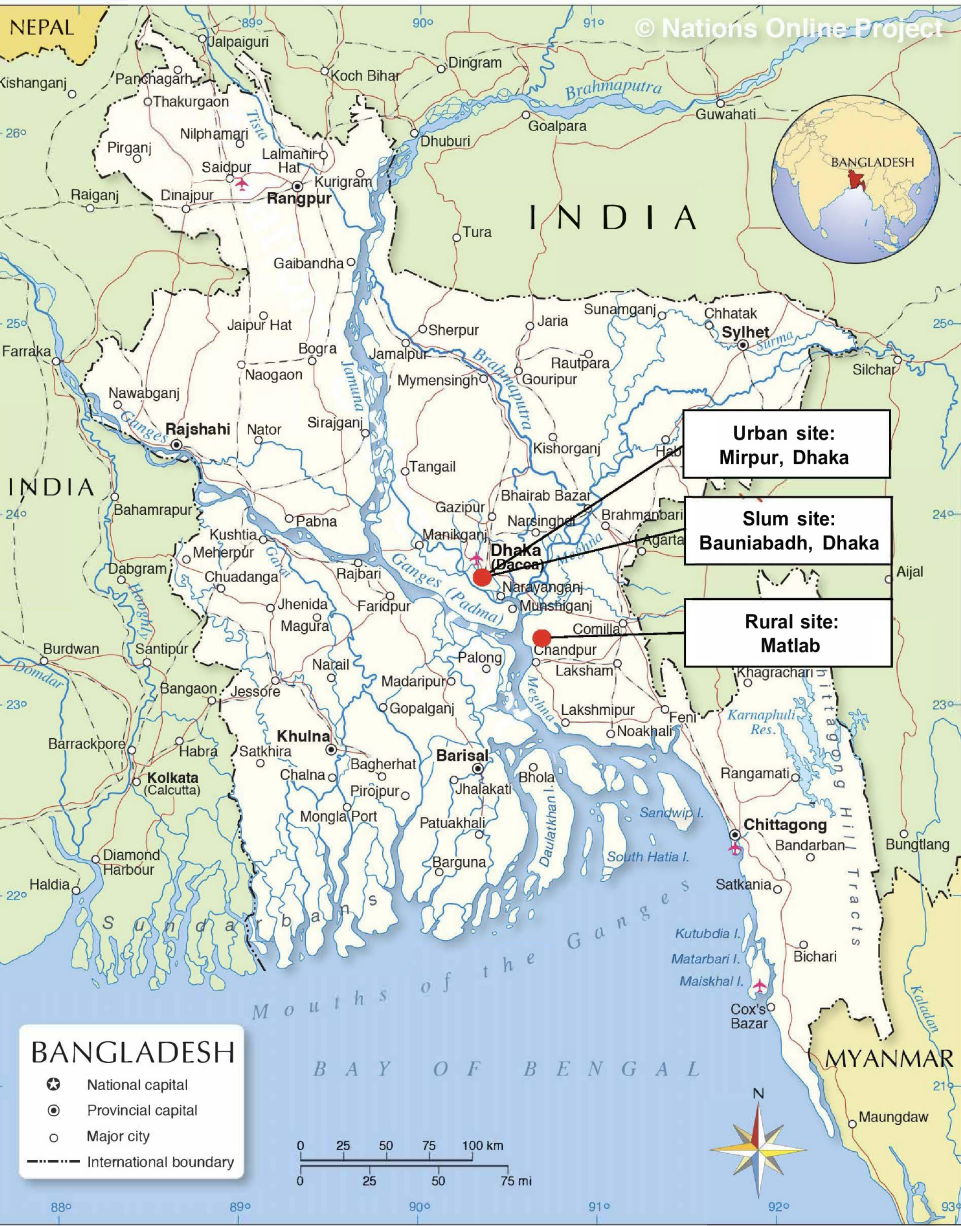
Location of the BELIEVE study sites. BELIEVE, BangladEsh Longitudinal Investigation of Emerging Vascular and nonvascular Events.

**Figure 2:**
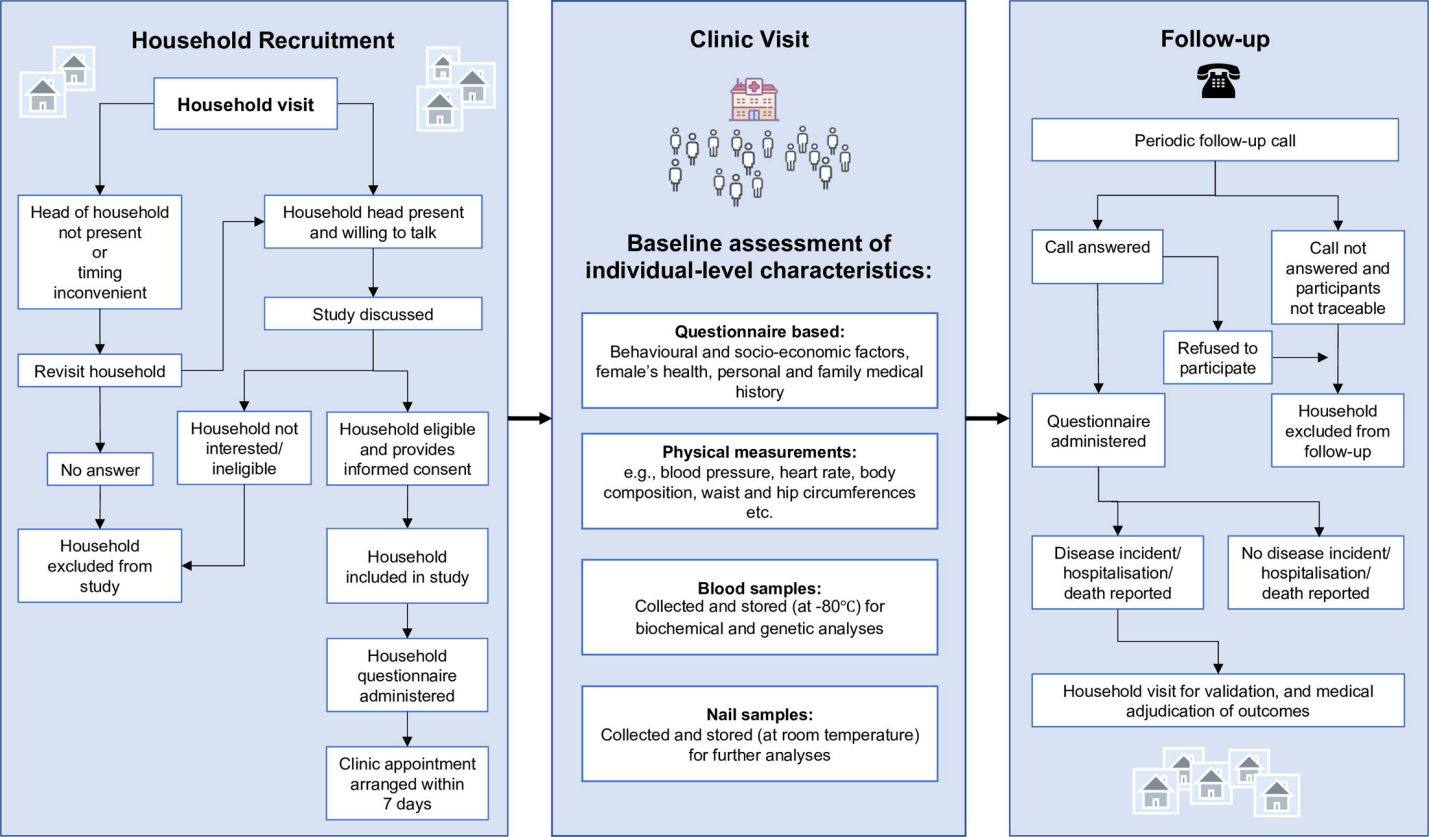
Study recruitment and follow-up procedures.

Between January 2016 and March 2020, the BELIEVE prospective cohort study recruited 73 883 participants from 30 817 households across three different settings in Bangladesh: urban (Mirpur-Dhaka), rural (Matlab-Chandpur) and urban-poor (‘slum’; Bauniabadh-Dhaka) (figure 1). Households were initially identified through complete surveys that had previously mapped households’ information across the three study sites. Individuals living in the identified households were eligible for enrolment into the study if they (1) were aged 11 years or older; (2) had lived in their current household for at least 3 years; (3) were intending to reside in the study site for at least a further 5 years and (4) were willing to provide written informed consent and prospective follow-up information. For all participants, the recruitment procedure first involved trained study personnel visiting all identified households to provide written and verbal information about the study. On completion of the household visit, all eligible household members were invited to attend a local study clinic to complete an individual-level baseline assessment (figure 2).

#### Questionnaire administration and physical measurements at baseline assessment

The study questionnaire was adapted to the Bangladesh context based on validated questionnaires used in previous large-scale studies.^12^,^14^ The initial version of the structured questionnaire underwent preliminary testing with a small group of individuals from the target population to evaluate clarity, relevance and cultural appropriateness. Following this, a larger group of individuals was involved for further validation and refinements, and adjustments were made to enhance readability and ensure logical flow, before finalising the questionnaire for the main study. To ensure both linguistic accuracy and cultural relevance, the questionnaire was translated into Bengali using a rigorous translation and back-translation process. During the household visit, trained personnel administered the structured questionnaire to collect household-level information and family structure characteristics (table 1) using a bespoke Android interface operated through handheld touchscreen tablet devices. During the clinic visit, trained personnel used a computer-based prepiloted epidemiological questionnaire to collect self-reported information on >300 items related to socioeconomic and demographic characteristics, consanguinity, behaviours (eg, tobacco and alcohol consumption, dietary intake, physical activity), female health, self-reported personal and family medical history and medication use (table 1; a copy of the study questionnaire is available in online supplemental appendix). Direct computer entry by participants, rather than interviews, was employed to enhance privacy when answering sensitive questions, with the option to skip these questions.

**Table 1:** Questionnaire-based information, physical measurements and biological samples collected at baseline in the BELIEVE study

Characteristics	Description
**House and household**	
House information	Location, type of accommodation, house construction materials, number of bedrooms, cooking source, sanitation, water source, indoor and outside environment
Household information	Number of occupants, contact details, family size
**Individual**	
Demographics	Age, sex, ethnicity, religion, marital status, consanguinity
Behavioural factors	Tobacco consumption (cigarette and non-cigarette), passive tobacco exposure, cooking habits, food frequency assessment, alcohol consumption, physical activity, sleeping habits, mobile phone usage
Socioeconomic factors	Education, occupation, income, remittances, loans, assets owned (including mobile phone, television, refrigerator, digital versatile disc player, air conditioning, bicycle, motorcycle, car, livestock, land, bank account)
Psychosocial factors	Stress at work and at home, social support, Centre for Epidemiological Studies Depression symptoms score, Generalised Anxiety Disorder score, sleeping habits, life events
Female health	Age at menarche, hormonal contraceptive usage, menstrual and pregnancy history
Personal and family medical history	Myocardial infarction, angina, hypertension, other vascular diseases, type 2 diabetes mellitus, atrial fibrillation, cancers, hypercholesterolaemia, chronic liver disease, chronic kidney disease, chronic obstructive respiratory disease, mental disorders, neurological diseases, infectious diseases, childhood disorders, major surgery, chest and limb pain, current medication usage
Physical measurements	Blood pressure, heart rate, respiratory rate, height, weight, body composition, upper body strength, waist and hip circumferences.
Biological samples	Serum, plasma and whole blood samples, finger and toe-nail clippings

BELIEVEBangladEsh Longitudinal Investigation of Emerging Vascular and nonvascular Events

Self-reported information on medical history and medication use was supplemented by medical records, drug prescriptions and by asking participants to bring their medications to the clinic visit. To assess local dietary patterns, benchmark food-frequency questionnaires previously developed and validated in Bangladesh were adapted for BELIEVE.^15^ These questionnaires estimated the standard portion size assigned to each food item and drinking water source. Participants’ phone numbers (or, for participants younger than 18 years, a contact number of a legal parent, guardian or caretaker) were routinely collected at the baseline visit for future contact and to collect follow-up information.

Standardised procedures and equipment were used to assess height, weight, waist and hip circumference, body composition, systolic and diastolic blood pressure, heart rate and upper body strength (online supplemental table 1). Briefly, blood pressure was measured using an automated device (Omron HEM 7130) on the right arm twice with a 3 min interval between each measurement, with the patient remaining in a sitting position for at least 5 minutes before the first evaluation. Height was measured using the ShorrBoard ICA Measuring Board to within 1 cm and weight was measured using the Tanita HD-661 scale to within 0.1 kg. Waist circumference was assessed using a non-stretchable soft standard measuring tape over the abdomen at the widest diameter between the costal margin and the iliac crest, and hip circumference at the level of the greater trochanters (ie, the widest diameter around the buttocks). Anthropometric measurements were performed in a standing position. Body composition was assessed by bioimpedance using the Tanita MC-780MA analyser and handgrip strength using a Jamar Plus Digital Hand Dynamometer.

To enhance consistency in the collection of data, we trained staff extensively and adopted standardised approaches, validated instruments and electronic data collection methods with built-in validity checks and queries. For example, the paper-free digital data collection platform involved extensive computerised checks to restrict missing values, duplications, inconsistencies and outliers. Information was transferred daily in a secure manner to the study’s central database at Cambridge University, with a copy also kept at the local recruitment centres for additional checks and queries. Reports were generated by data managers and reviewed by study Principal Investigators (PIs) and coordinators on a regular basis.

#### Collection, storage and initial use of biological samples

Participants provided non-fasting blood samples for research with the time of the last meal recorded. Up to 23 mL of non-fasted whole blood samples were collected from each participant aged 18 years and above in two tubes (11 mL EDTA and 12 mL serum). For participants aged 11–17 years, 12 mL of non-fasted whole blood samples were collected in two tubes (6 mL EDTA and 6 mL serum). Samples were centrifuged within 45 min of venipuncture (at 6000 rpm for 15 min). Isolated serum, EDTA plasma and whole blood samples were stored at −20°C before being transferred to a −86°C freezer at the end of each working day (online supplemental figure 1). To enable the measurement of additional potential risk factors (eg, metal contaminants), finger-nail and toe-nail clippings were collected in a subset of participants, kept separately in plastic bottles and stored at room temperature. Biological samples have been stored in long-term dual repositories located in Bangladesh and the UK to enable further assays. A range of biochemical and genomic analyses on the stored samples is currently ongoing.

#### Follow-up procedure and outcome ascertainment

Study participants are being actively followed up indefinitely for cause-specific mortality and incidence of selected non-fatal health outcomes (figure 2). Follow-up involves contact with participants by the study team at regular intervals (eg, every 18–24 months), during which trained personnel use an electronic questionnaire to collect information on medical events reported since the last contact. Based on the responses to the questionnaire (or if phone contact is unsuccessful), a household visit is scheduled to collect additional information and medical documents. During the household visit, trained personnel collect further information for selected major categories of non-fatal events (including myocardial infarction, diabetes, stroke and common cancers) using a structured questionnaire and a bespoke Android electronic interface operated through handheld touchscreen tablet devices.

As the majority of individuals in the study settings who have received medical attention tend to have paper copies of medical documents in their homes, study personnel visiting households collect digital photographs of any relevant medical documents available (including discharge summaries, diagnostic test reports, medications and death certificates) using tablet devices. If medical documents are unavailable, written informed consent is obtained to retrieve these from the relevant hospitals. Nearly all deaths in the study areas will have involved some form of medical attention, with their underlying causes certified by a medical doctor. Verbal autopsy is conducted in all deaths by trained personnel to help determine the most likely cause from symptoms or signs described by family members.^16^ To help adjudicate and classify health outcomes (eg, into definite, probable and possible categories), the information collected described above is periodically reviewed by medically trained personnel.

The quality and completeness of both mortality and morbidity follow-up data in each study site are regularly checked during the study period by the study coordinating centres. This involves monitoring the number of people who have died or are lost to follow-up, assessment of overall mortality patterns in the study cohort, levels of diagnostic criteria for individual diseases and the proportion of deaths with unknown causes.

#### Patient and public involvement

In keeping with this study’s principles of cocreation and community engagement, we have implemented a participatory strategy, collaborating closely with local residents and key stakeholders (some of whom were study participants). Community consultations were conducted in communal spaces to communicate the study’s objectives and actively seek feedback from community members. Invitations were extended to local leaders for these sessions, facilitating a deeper understanding of the community’s needs and concerns and allowing us to tailor the study accordingly. In addition, we employed local community members as field team personnel, who were trained to collect data and assist study participants. This approach not only provided employment opportunities for local people but also enhanced the cultural appropriateness of the study approaches we used. As well as cocreating the study design, study participants were provided information about the study’s purpose, with the assurance that their information would be exclusively used for research purposes.

#### Sample size considerations and statistical analysis

Sample size considerations have been guided by a combination of pragmatic constraints (eg, the availability of resources) and statistical power calculations. Assuming 5% of the population develops a disease condition during follow-up, a study of 70 000 individuals should provide 80% power to detect an HR of about 1.1 per 1 SD higher value at 5% significance level. In this report, continuous variables were summarised as mean (SD) or median and IQR, and categorical variables were summarised as frequencies and percentages. A complete case analysis was used to handle missing data. Variables were summarised and compared across relevant population subgroups (eg, study site, sex and age group) using the t-test for continuous variables and χ^2^ test for categorical variables. The prevalences of chronic conditions at baseline were summarised as overall crude prevalences, and by site, sex and 20-year age group. To make comparisons between sites analysis was conducted restricted to data from participants aged between 20 and 79 years to compare prevalences of conditions standardised according to the Bangladesh 2022 population structure.^17^ All statistical tests were two sided, and the significance level was set at p<0.05. Robust SEs were estimated to allow for the clustering of participants by household. Analyses were performed using STATA (V.16, StataCorp) and R (V.4.0.1, R Foundation for Statistical Computing). Future statistical analyses will be developed following relevant guidelines for cohort data with household clustering (eg, Strengthening the Reporting of Observational Studies)^18^ and will be presented elsewhere.

### Findings to date

#### Demographic, behavioural and physical characteristics in the overall study population

Overall, 59 846 (81%) participants were recruited from Mirpur-Dhaka (urban site), 5332 (7%) from Bauniabadh-Dhaka (slum site) and 8705 (12%) from Matlab-Chandpur (rural site). 43 470 (59%) participants were female; the mean (SD) age at recruitment was 39 (15) years; 4122 participants (5.6%) were aged between 11 and 17 years inclusive (table 2). At the time of the baseline survey, 38 848 (52%) participants had either no formal education or primary level education only; 17% were current smokers; 23% were current users of chewable tobacco products and only 1.3% of the participants reported consuming alcohol regularly.

**Table 2:** Baseline characteristics of the 73 833 BELIEVE study participants

Key characteristics	Mean (SD) ornumber (%)
Demographic and behavioural characteristics	
Age (years)	39 (15)
Individuals aged <18 years	4122 (5.6)
Female	43 470 (59)
Study site of residence	
Urban site	59 846 (81)
Urban slum site	5332 (7.2)
Rural site	8705 (12)
Married	54 386 (74)
Education level	
None/preprimary	16 594 (22)
Primary	22 254 (30)
Secondary	21 367 (29)
Bachelors or higher degree	13 105 (18)
Current smoker	12 450 (17)
Current chewable tobacco user	16 982 (23)
Current alcohol consumer	927 (1.3)
Physical measurements	
Systolic blood pressure (mm Hg)	123 (21)
Diastolic blood pressure (mm Hg)	77 (12)
Body mass index (BMI, kg/m^2^)	25 (5)
Waist circumference (cm)	86 (12)
Waist to hip ratio	0.92 (0.08)
Body fat percentage (%)	27 (9)

Missing data by variable: Married (n=3), Eeducation (n=563), smoking/chewable tobacco use (n=4), Aalcohol consumption (n=42), systolic blood pressure (n=23), diastolic blood pressure (n=22), BMI (n=45), waist circumference (n=26), waist to hip ratio (n=30).

Body fat percentage was not measured in the early stages of recruitment and therefore was not available in 15 049 participants.

BELIEVEBangladEsh Longitudinal Investigation of Emerging Vascular and nonvascular Events

Several characteristics varied by study site. For example, 55% of participants in the urban site had a secondary or higher level of education compared with only 16% and 18% in slum and rural sites, respectively (online supplemental table 2). Whereas the percentages of participants self-reporting as current smokers were similar across the sites (17%, 17% and 13% in urban, slum and rural sites, respectively), reported use of chewable tobacco and alcohol was higher in the slum site (62% and 12%, respectively, vs 18% and 0.4% in the urban site, and 34% and 0.4% in the rural site). Additional conventional risk factor values differed by study site, with a tendency towards more adverse risk factor profiles in urban participants (online supplemental table 2). For example, mean (SD) systolic blood pressure was 125 (21) mm Hg in participants from the urban site vs 112 (20) and 120 (20) mm Hg in the slum and rural sites, respectively. Mean (SD) body mass index (BMI) was 26 (5) kg/m^2^, 23 (5) kg/m^2^ and 23 (5) kg/m^2^ in participants from the urban, slum and rural sites, respectively, with other measures of adiposity (including, waist-to-hip ratio and body fat percentage) following a similar trend. As the trend towards urbanisation accelerates in Bangladesh, such differences in risk factor profiles portend implications for striking increases in NCDs.^19 20^ This contrast in risk factor profiles across settings also suggests the need for context-specific solutions to help better prevent and control NCDs. We plan to contribute towards this goal through longitudinal surveillance of risk factors and NCD incidence in the BELIEVE cohort, as well as the repurposing of the cohort framework to support applied health research studies.

Certain baseline characteristics also differed between males and females (online supplemental table 3). Females were, on average, younger (38 vs 41 years), had spent fewer years in education (45% vs 50% had a secondary or higher level of education), were less likely to report any tobacco smoking (<1% vs 41%) and consuming alcohol (<1% vs 3%). Systolic blood pressure was higher among males (127 vs 121 mm Hg), whereas females had a higher BMI (26 vs 24 kg/m^2^).

#### Overweight, obesity and chronic health conditions in adults aged 20–79

Among adult participants aged between 20 and 79 years (online supplemental table 4–6) and using WHO-defined BMI categories, 39% of individuals were considered overweight (BMI 25–29 kg/m^2^) and 16% obese (ie, BMI≥30 kg/m^2^ online supplemental table 4). After standardisation of the sex and age distribution of the Bangladeshi population for the same age range, the corresponding proportions were 37% and 14%, respectively (online supplemental figure 2). Proportions of overweight and obese participants were higher in the urban site, compared with the slum and rural sites, and also higher in females compared with males (online supplemental figure 2–3). There are likely to be multiple factors contributing to excess adiposity, including dietary patterns, sedentary lifestyles and urbanisation.^1 21^ Additionally, socioeconomic disparities and lack of awareness regarding the importance of diet and physical activity may play roles. As overweight and obesity are associated with multiple chronic health conditions, including cardiovascular diseases, diabetes and certain cancers,^22^,^24^ addressing this challenge is necessary to improve the health of the Bangladeshi population and alleviate healthcare system burdens.^25 26^

**Figure 3:**
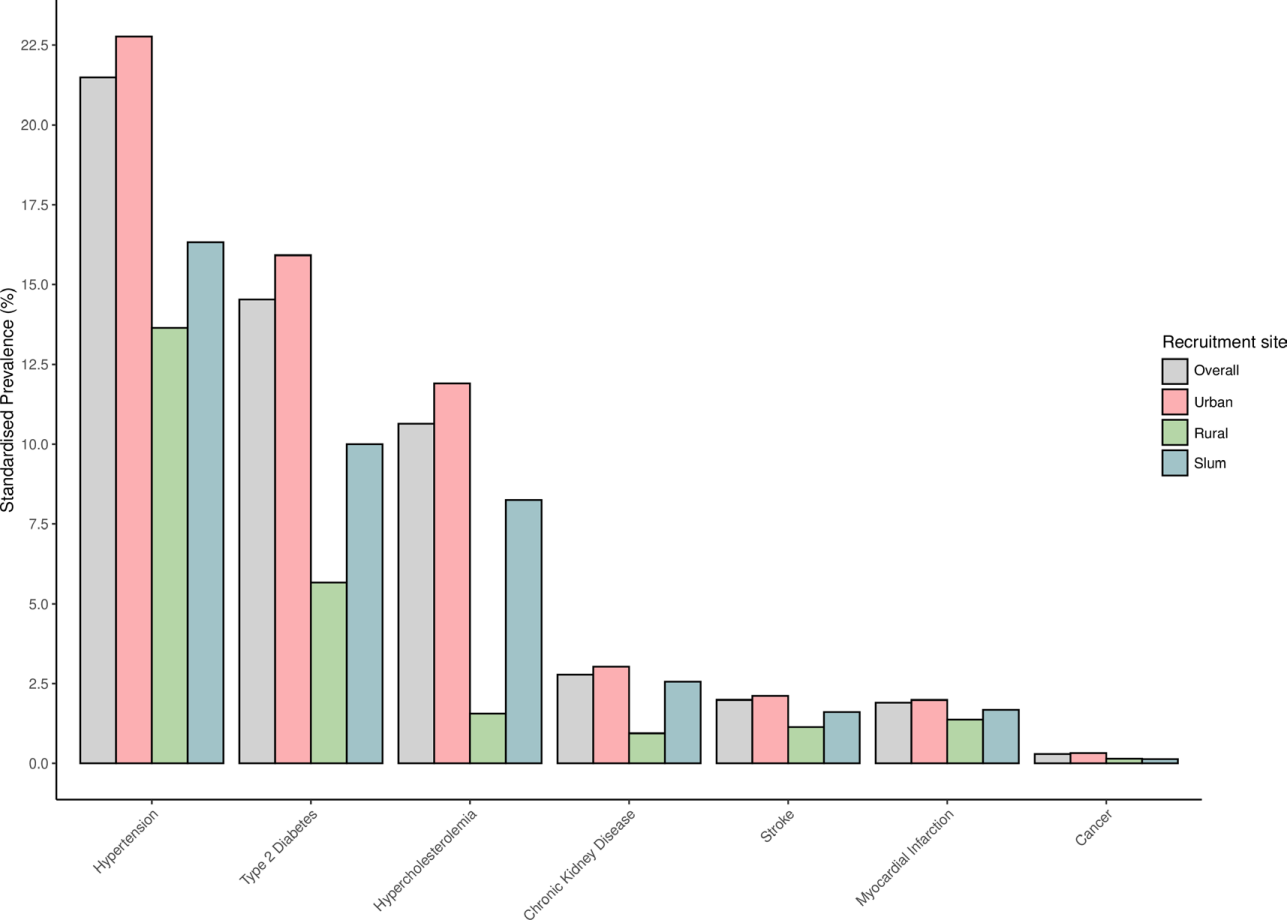
Standardised prevalences of selected chronic conditions at recruitment in BELIEVE for participants aged 20–79. Prevalence was standardised using the sex and age distribution of the Bangladesh 2022 population aged 20–79. BELIEVE, BangladEsh Longitudinal Investigation of Emerging Vascular and nonvascular Events.

The most prevalent self-reported chronic health conditions recorded at baseline among adults aged 20–79 were hypertension, type 2 diabetes and hypercholesterolaemia (online supplemental table 6), with standardised prevalences of 21%, 15% and 11%, respectively (figure 3). The prevalence of these conditions was higher among participants recruited in the urban site compared with those in slum and rural sites. For example, the standardised prevalence of hypertension was 23%, 16% and 14% in urban, slum and rural sites, respectively. Corresponding prevalences were 16%, 10% and 5.7% for type 2 diabetes, and 12%, 8% and 1.5% for hypercholesterolaemia. The overall prevalence of type 2 diabetes in this study was higher than those previously reported for Bangladesh^19^ and varied by age and sex, with the highest prevalences among older participants and females (online supplemental figure 4–6). These findings may suggest potential age-specific and sex-specific differences in susceptibility to type 2 diabetes or disparities in healthcare access and management or both. We plan to contribute towards understanding such potential age-specific and sex-specific differences in determinants of chronic diseases, as it should inform the development of targeted preventive measures and tailored interventions to address the specific risk factors affecting different populations.

### Strengths and limitations

Although Bangladesh is experiencing substantial increases in NCDs,^7^ there is limited evidence about the multidimensional risk factors and drivers of NCDs in the country, thereby preventing a deeper understanding of the web of causation for NCDs and limiting development of optimal prevention and control approaches. The BELIEVE study has been established as a long-term epidemiological bioresource to help address this gap, including an investigation of Bangladesh’s contrasting urban and rural settings. Configured to investigate biological, behavioural, environmental and broader determinants of NCDs, the BELIEVE study is a household-based prospective cohort study that spans urban, slum and rural settings. To our knowledge, it represents the largest bioresource for NCDs and related traits in Bangladesh, involving active longitudinal follow-up of participants for new-onset health outcomes.

The BELIEVE study has demonstrated the feasibility of employing modern epidemiological methods at scale in a population-based study located across multiple different settings in Bangladesh. The study has used electronic data collection methods, using study forms implemented through bespoke software applications (‘apps’), allowing comprehensive recording of questionnaire-based data, as well as physical measurements. The study has also collected a range of biological samples, including serum, plasma, whole blood and nail samples stored in long-term repositories. The assay of these samples (which already includes genotyping, sequencing, multiple molecular ‘omics, and soluble clinical biomarkers) enables the study of many molecular factors, laying the foundations for an advanced understanding of disease pathways and potential therapeutic targets for the treatment and prevention of NCDs in Bangladesh and beyond.

The potential limitations of the BELIEVE study merit consideration. Within each of the three study settings, every household was invited to participate in the cohort. As the household participation rates have been high (>80%), they suggest that study participants should be broadly representative of the source population in the participating sites. However, the participants, households and sites included in the study were not necessarily representative of the general Bangladesh population. Nevertheless, it should be reasonable to infer that findings from this cohort can be generalised to other similar settings in Bangladesh because the study involves participants with a wide range of diverse characteristics (eg, in relation to age, sex, socioeconomic status, education level and occupation), recruited from a variety of different settings (urban, slum and rural areas) characteristic of contemporary Bangladesh.

A further potential limitation relates to the scope for misclassification of risk factors and health outcomes recorded at baseline and during study follow-up because of inaccuracies in participant self-reports. To help limit the effects of such potential misclassification, the BELIEVE study is supplementing self-reported data with the use of objective measurements (eg, assay of low-density lipoprotein -cholesterol (LDL-C), glycated haemoglobin (HbA1c) and arsenic metabolites), inspection of health records kept by participants during household follow-up visits, use of previously validated ‘verbal autopsy’ methods, and exploration of emerging potential linkages of study participants with digital health records kept at community healthcare and hospital levels.

Finally, there is potential in this prospective study for underestimation of the strength of the associations observed between risk factors and incident health outcomes due to fluctuations in risk factor levels within individuals over time (ie, ‘regression dilution’ bias). To help limit such bias, the BELIEVE study plans to conduct periodic resurveys of risk factor levels in randomly selected subsets of the study participants.

### Collaboration

We welcome potential collaboration with other researchers. Data are available on application to the study’s Steering and Data Access Committee.

## Conclusion

By providing a powerful resource to investigate multidimensional determinants of NCDs across diverse settings in Bangladesh, the BELIEVE study should help advance scientific understanding and inform public health action in an archetypal LMIC with pressing public health needs.

## supplementary material

10.1136/bmjopen-2024-088338 online supplemental file 1

## Data Availability

Data are available on reasonable request.
