# Supplementary material for: Cohort profile: the BangladEsh Longitudinal Investigation of Emerging Vascular and nonvascular Events (BELIEVE) cohort study
Source: BMJ Open. 2025 Jan 22;15(1):e088338. doi: 10.1136/bmjopen-2024-088338 (PMC11784369; doi:10.1136/bmjopen-2024-088338)
Supplement: online supplemental file 1 [file bmjopen-15-1-s001.docx]

**Supplementary Material**

**Supplementary Table 1**. A detailed description of physical measurements in the BELIEVE study

| **Test** | **Description** |
| --- | --- |
| **Blood Pressure and Heart Rate** | Blood pressure and heart rate were measured using an automated upper arm device. Measurements were taken after a period of 5 minutes during which the participant was sitting quietly; i) in a seated position with legs uncrossed and feet flat on the floor; ii) with the appropriate cuff size placed snugly around the upper arm, leaving a space for a finger between the participant and cuff; iii) ensuring that the cuff was appropriately aligned with the brachial artery following the instructions in the equipment manual. Two measurements of blood pressure and heart rate were taken. |
| **Height** | Standing height was measured using a stadiometer standardised across all study sites to the nearest 1cm. Participants were asked to remove their shoes before the measurement being taken and to stand upright with heels and shoulders against the measuring rod, knees and back straight and looking forward. The evaluating arm was pushed down to lightly touch the participant’s head. Two measurements of height were taken. |
| **Weight** | Weight was measured using a device standardised across all study sites to the nearest 0.1kg. The scales were standardised to 0 before each use. Weight was measured in light clothing; participants were asked to remove their outer garments (e.g., coats) and shoes. Participants were asked to stand on the centre of the scales, arms to the side, looking forward. Two measurements of weight were taken. |
| **Waist Circumference** | Waist circumference was measured with the participant standing, using equipment standardised across study sites. This was measured to the nearest 0.1 cm using a non-stretchable standard tape measure. Subject was relaxed with arms held loosely at sides. Waist circumference was measured over the abdomen at the widest diameter between the costal margin and the iliac crest over light clothing. Two measurements were taken for waist circumference. |
| **Hip Circumference** | Hip circumference was measured, with the participant standing, using equipment standardised across study sites. This was measured to the nearest 0.1 cm using a non-stretchable standard tape measure. Subject should relax with arms held loosely at sides. Hip circumference was measured at the widest diameter around the buttocks. The tape measure must be kept horizontal for standing measurement. Two measurements were taken for hip circumference. |
| **Respiratory Rate** | Respiratory rate was measured with the participant seated. The Field Research Assistants counted the number of breaths per minute by careful observations without the participant being aware. This was timed with for 60 seconds with a watch. |
| **Body Composition** | Body Composition was calculated by measuring bio-impedance using the Tanita MC-780MA body composition analyser. Participants were directed and monitored during use of this machine by trained study clinic nurses, following the standard operating procedures (appendix 5). |
| **Upper Body Strength** | Upper body strength was measured using a Jamar Plus Digital Hand Dynamometer. Trained FRA’s monitored and directed participants in the correct usage of the handgrip dynamometer (detailed in appendix 7). |

**Supplementary Table 2:** Baseline characteristics of the BELIEVE study participants by site

| Characteristics | Urban | | Urban Slum | | Rural | |
| --- | --- | --- | --- | --- | --- | --- |
|  | **Number of available observations** | **Mean (SD) or Number (%)** | **Number of available observations** | **Number (%)** | **Number of available observations** | **Mean (SD) or Number (%)** |
|  |  |  |  |  |  |  |
| **Demographic and behavioural characteristics** |  |  |  |  |  |  |
| Age (years) | 59846 | 40 (15) | 5332 | 32 (15) | 8705 | 37 (18) |
| Individuals aged <18 years |  | 968 (1.6) |  | 1232 (23) |  | 1922 (22) |
| Females | 59846 | 34780 (58) | 5332 | 3372 (63) | 8705 | 5318 (61) |
| Married | 59844 | 45332 (76) | 5331 | 3309 (62) | 8705 | 5745 (66) |
| Education level | 59368 |  | 5310 |  | 8642 |  |
| None/pre-primary |  | 9808 (17) |  | 2831 (53) |  | 3955 (46) |
| Primary |  | 17449 (29) |  | 1630 (31) |  | 3175 (37) |
| Secondary |  | 19319 (33) |  | 771 (15) |  | 1277 (15) |
| Bachelors or higher degree |  | 12792 (22) |  | 78 (1) |  | 235 (3) |
| Current smoker | 59844 | 10363 (17) | 5331 | 913 (17) | 8704 | 1174 (13) |
| Current chewable tobacco user | 59843 | 10734 (18) | 5331 | 3322 (62) | 8705 | 2926 (34) |
| Current alcohol consumer | 59820 | 244 (0.4) | 5324 | 650 (12) | 8697 | 33 (0.4) |
|  |  |  |  |  |  |  |
| **Physical measurements** |  |  |  |  |  |  |
| Systolic blood Pressure (mmHg) | 59831 | 125 (21) | 5324 | 112 (20) | 8705 | 120 (20) |
| Diastolic blood Pressure (mmHg) | 59832 | 78 (11) | 5324 | 70 (12) | 8705 | 74 (12) |
| Body Mass Index (BMI, kg/m^2^) | 59815 | 26 (5) | 5323 | 23 (5) | 8700 | 23 (5) |
| Waist circumference (cm) | 59828 | 87 (12) | 5324 | 83 (14) | 8705 | 80 (13) |
| Waist to hip ratio | 59824 | 0.93 (0.08) | 5324 | 0.91 (0.08) | 8705 | 0.91 (0.08) |
| Body fat percentage (%) | 45432 | 28 (9) | 5140 | 27 (9) | 8262 | 24 (9) |
|  |  |  |  |  |  |  |

**Supplementary Table 3**: Baseline characteristics of the BELIEVE study participants by sex

| Characteristics | Females | | Males | |
| --- | --- | --- | --- | --- |
|  | **Number of available observations** | **Mean (SD) or Number (%)** | **Number of available observations** | **Mean (SD) or Number (%)** |
|  |  |  |  |  |
| **Demographic and behavioural characteristics** |  |  |  |  |
| Age (years) | 43470 | 38 (15) | 30413 | 41 (16) |
| Individuals aged <18 years |  | 2307 (5.3) |  | 1815 (6.0) |
| Study site of residence | 43470 |  | 30413 |  |
| Urban |  | 34780 (80) |  | 25066 (82) |
| Urban slum |  | 3372 (7.8) |  | 1960 (6.4) |
| Rural |  | 5318 (12) |  | 3387 (11) |
| Married | 43467 | 32082 (74) | 30413 | 22304 (73) |
| Education level | 43129 |  | 30191 |  |
| None/pre-primary |  | 10412 (24) |  | 6182 (20) |
| Primary |  | 13406 (31) |  | 8848 (29) |
| Secondary |  | 12334 (29) |  | 9033 (30) |
| Bachelors or higher degree |  | 6977 (16) |  | 6128 (20) |
| Current Smoker | 43466 | 63 (0) | 30413 | 12387 (41) |
| Current Chewable tobacco user | 43467 | 10376 (24) | 30412 | 6606 (22) |
| Current Alcohol consumer | 43442 | 122 (0.3) | 30399 | 805 (2.6) |
|  |  |  |  |  |
| **Physical measurements** |  |  |  |  |
| Systolic Blood Pressure (mmHg) | 43458 | 121 (21) | 30402 | 127 (20) |
| Diastolic Blood Pressure (mmHg) | 43458 | 76 (12) | 30403 | 78 (12) |
| Body Mass Index (BMI, kg/m^2^) | 43447 | 26 (5) | 30391 | 24 (4) |
| Waist Circumference (cm) | 43457 | 87 (13) | 30400 | 86 (12) |
| Waist to hip ratio | 43455 | 0.91 (0.08) | 30398 | 0.94 (0.07) |
| Body Fat Percentage (%) | 34735 | 32 (7) | 24099 | 20 (6) |
|  |  |  |  |  |

**Supplementary Table 4.** Baseline characteristics of the BELIEVE study participants aged 20-79, excluding pregnant females

| Characteristics | Number of available observations | Mean (SD) or  Number (%) |
| --- | --- | --- |
|  |  |  |
| **Demographic and behavioural characteristics** |  |  |
| Age (years) | 65199 | 42 (14) |
| Females | 65199 | 38196 (59) |
| Study site of residence | 65199 |  |
| Urban site |  | 54833 (84) |
| Urban slum site |  | 3901 (6.0) |
| Rural site |  | 6465 (9.9) |
| Married | 65196 | 52459 (80) |
| Education level | 64709 |  |
| None/pre-primary |  | 13888 (21) |
| Primary |  | 19268 (30) |
| Secondary |  | 19260 (30) |
| Bachelors or higher degree |  | 12293 (19) |
| Current smoker | 65195 | 11767 (18) |
| Current chewable tobacco user | 65195 | 15962 (24) |
| Current alcohol consumer | 65167 | 722 (1.1) |
|  |  |  |
| **Physical measurements** |  |  |
| Systolic blood pressure (mmHg) | 65181 | 125 (21) |
| Diastolic blood pressure (mmHg) | 65182 | 78 (11) |
| Body Mass Index (BMI, kg/m^2^) | 65163 | 26 (5) |
| Waist circumference (cm) | 65179 | 88 (11) |
| Waist to hip ratio | 65175 | 0.93 (0.07) |
| Body fat percentage (%) | 51846 | 28 (9) |
|  |  |  |
| **WHO-defined BMI categories** |  |  |
| Underweight (<18.5 kg/m^2^) |  | 3145 (4.8) |
| Normal weight (18.5-24.9 kg/m^2^) |  | 26074 (40) |
| Overweight (25.0-29.9 kg/m^2^) |  | 25502 (39) |
| Obese (≥30 kg/m^2^) |  | 10442 (16) |
|  |  |  |

**Supplementary Table 5:** Baseline characteristics of the BELIEVE study participants by age

| Characteristics | Age 11-19 years | | Age 20-39 years | | Age 40-59 years | | Age 60-79 years | | Age >=80years | | |
| --- | --- | --- | --- | --- | --- | --- | --- | --- | --- | --- | --- |
|  | **Number of available observations** | **Mean (SD) or Number (%)** | **Number of available observations** | **Mean (SD) or Number (%)** | **Number of available observations** | **Mean (SD) or Number (%)** | **Number of available observations** | **Mean (SD) or Number (%)** | | **Number of available observations** | **Mean (SD) or Number (%)** |
|  |  |  |  |  |  |  |  |  |  | |  |
| **Demographic and behavioural characteristics** | |  |  |  |  |  |  |  |  | |  |
| Age (years) | 7744 | 16 (3) | 32780 | 30 (6) | 25258 | 49 (6) | 7784 | 66 (5) | 317 | | 84 (4) |
| Females | 7744 | 4546 (59) | 32780 | 20943 (64) | 25258 | 14442 (57) | 7784 | 3434 (44) | 317 | | 105 (33) |
| Married | 7744 | 1127 (15) | 32779 | 25072 (76) | 25257 | 22449 (89) | 7783 | 5558 (71) | 317 | | 180 (57) |
| Education level | 7680 |  | 32541 |  | 25077 |  | 7711 |  | 311 | |  |
| None/pre-primary |  | 2499 (33) |  | 6543 (20) |  | 5718 (23) |  | 1748 (23) |  | | 86 (28) |
| Primary |  | 2711 (35) |  | 10544 (32) |  | 7133 (28) |  | 1784 (23) |  | | 82 (26) |
| Secondary |  | 1818 (24) |  | 10008 (31) |  | 7313 (29) |  | 2138 (28) |  | | 90 (29) |
| Bachelors or higher degree |  | 652 (8) |  | 5446 (17) |  | 4913 (20) |  | 2041 (26) |  | | 53 (17) |
| Current Smoker | 7744 | 653 (8) | 32778 | 6258 (19) | 25257 | 4504 (18) | 7783 | 1006 (13) | 317 | | 29 (9) |
| Current Chewable tobacco user | 7744 | 823 (11) | 32778 | 4146 (13) | 25257 | 8300 (33) | 7783 | 3552 (46) | 317 | | 161 (51) |
| Current Alcohol consumer | 7735 | 205 (2.7) | 32763 | 516 (1.6) | 25249 | 191 (0.8) | 7777 | 15 (0.2) | 317 | | 0 (0.0) |
|  |  |  |  |  |  |  |  |  |  | |  |
| **Physical measurements** |  |  |  |  |  |  |  |  |  | |  |
| Systolic Blood Pressure (mmHg) | 7739 | 107 (13) | 32771 | 116 (15) | 25251 | 131 (21) | 7782 | 143 (23) | 317 | | 152 (24) |
| Diastolic Blood Pressure (mmHg) | 7739 | 66 (9) | 32771 | 75 (10) | 25252 | 82 (11) | 7782 | 81 (11) | 317 | | 79 (12) |
| Body Mass Index (BMI, kg/m^2^) | 7735 | 21 (4) | 32763 | 25 (5) | 25244 | 26 (4) | 7779 | 25 (4) | 317 | | 23 (4) |
| Waist Circumference (cm) | 7738 | 71 (11) | 32770 | 85 (11) | 25250 | 91 (10) | 7782 | 91 (11) | 317 | | 89 (11) |
| Waist to hip ratio | 7738 | 0.84 (0.06) | 32768 | 0.90 (0.07) | 25248 | 0.96 (0.06) | 7782 | 0.98 (0.07) | 317 | | 0.99 (0.07) |
| Body Fat Percentage (%) | 6742 | 23 (8) | 25884 | 27 (9) | 20082 | 29 (9) | 5898 | 27 (9) | 228 | | 25 (8) |
|  |  |  |  |  |  |  |  |  |  | |  |

**Supplementary Table 6.** Crude prevalence of selected chronic conditions at recruitment in BELIEVE participants aged 20-79, overall and by site

| Chronic condition present at baseline | Overall | |  | Urban | | Urban Slum | | Rural | |
| --- | --- | --- | --- | --- | --- | --- | --- | --- | --- |
|  | **Number of available observations** | **Number (%) with condition** |  | **Number of available observations** | **Number (%) with condition** | **Number of available observations** | **Number (%) with condition** | **Number of available observations** | **Number (%) with condition** |
|  |  |  |  |  |  |  |  |  |  |
|  |  |  |  |  |  |  |  |  |  |
| Hypertension | 65819 | 15411 (23) |  | 55378 | 13795 (25) | 6541 | 1064 (16) | 3900 | 552 (14) |
| Type 2 diabetes | 65821 | 10578 (16) |  | 55379 | 9776 (18) | 6541 | 452 (7) | 3901 | 350 (9) |
| Hypercholesterolemia | 65820 | 7624 (12) |  | 55379 | 7241 (13) | 6541 | 110 (2) | 3900 | 273 (7) |
| Stroke | 65820 | 1371 (2.1) |  | 55379 | 1232 (2.2) | 6541 | 90 (1.4) | 3900 | 49 (1.3) |
| Myocardial infarction | 65820 | 1332 (2.0) |  | 55379 | 1178 (2.1) | 6541 | 109 (1.7) | 3900 | 45 (1.2) |
| Chronic kidney disease | 65819 | 1928 (2.9) |  | 55378 | 1764 (3.2) | 6541 | 66 (1.0) | 3900 | 98 (2.5) |
| Cancer | 65818 | 220 (0.33) |  | 55379 | 204 (0.37) | 6540 | 10 (0.15) | 3899 | 6 (0.15) |
|  |  |  |  |  |  |  |  |  |  |

**Supplementary Figure 1.** Standard Operating Procedures – Blood Sample Collection, Labelling and Processing

**Supplementary Figure 2.** Standardised prevalences of WHO-defined BMI categories in BELIEVE participants aged 20-79, overall and by site


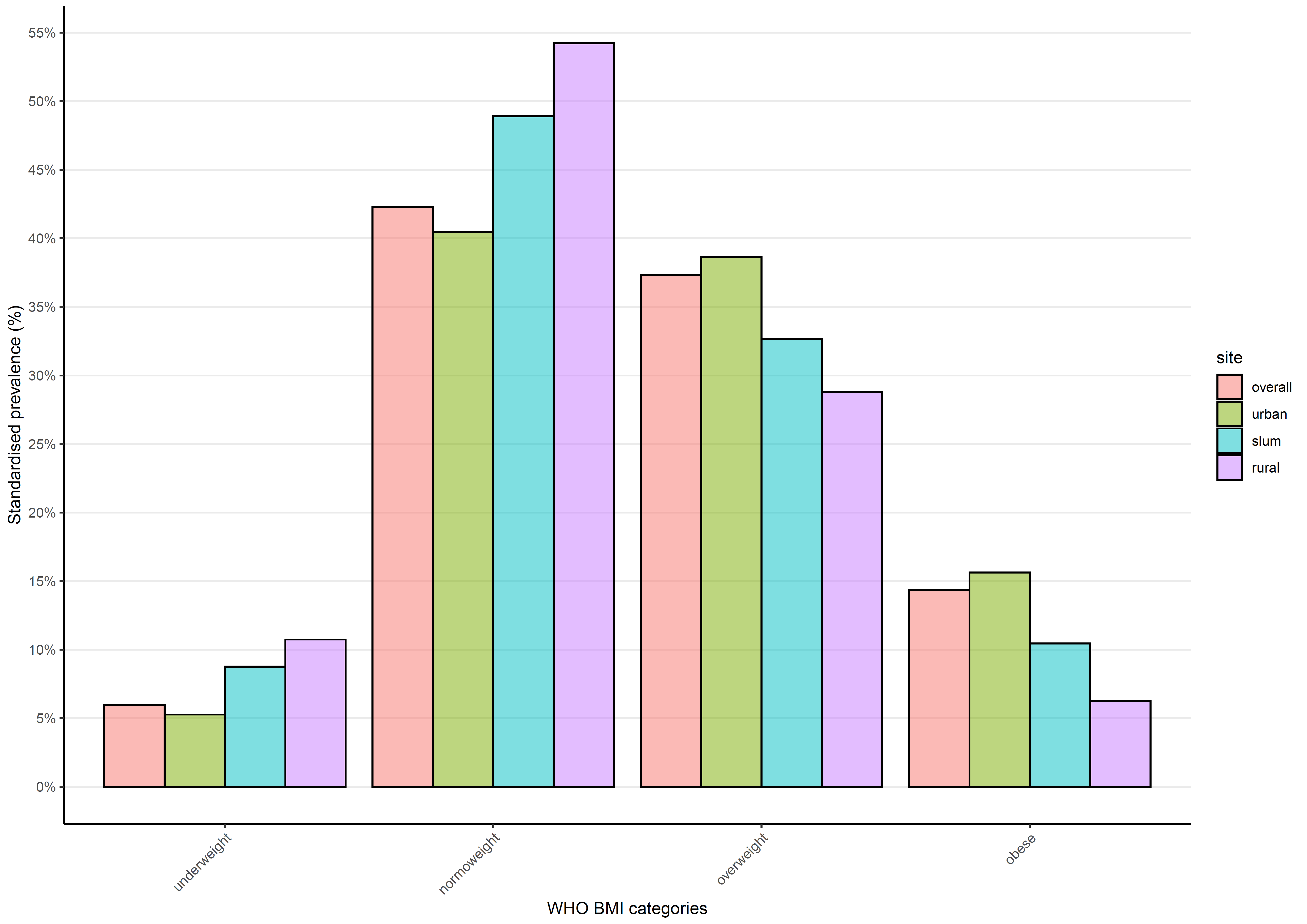


Prevalence is standardised using the sex and age distribution of the Bangladesh 2022 population aged 20-79

**Supplementary Figure 3.** Standardised prevalences of WHO-defined BMI categories in BELIEVE participants aged 20-79, by sex and site


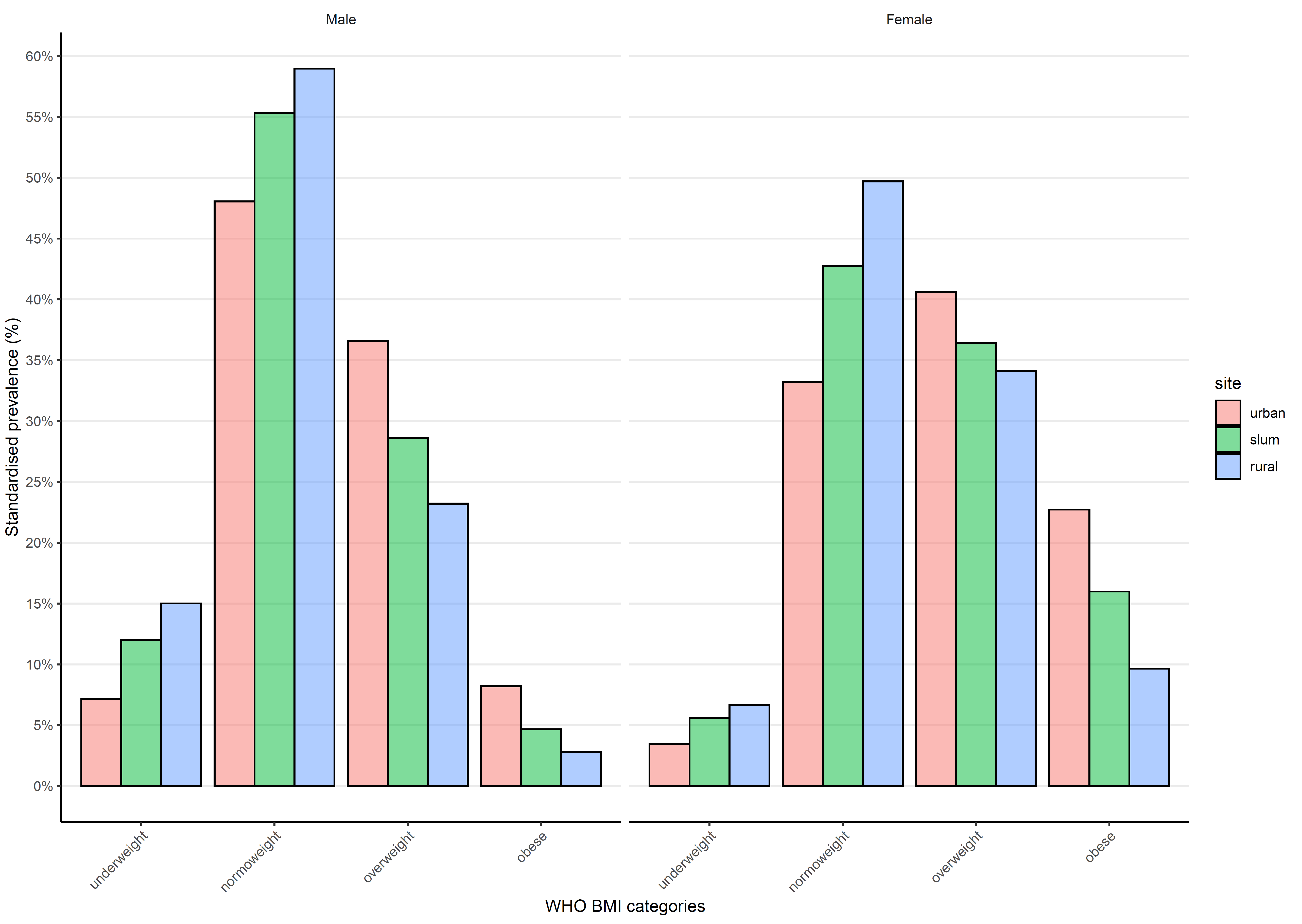


Prevalence was standardised using the sex and age distribution of the Bangladesh 2022 population aged 20-79.

**Supplementary Figure 4.** Standardised prevalence of selected chronic conditions in participants aged 20-79, by age and site


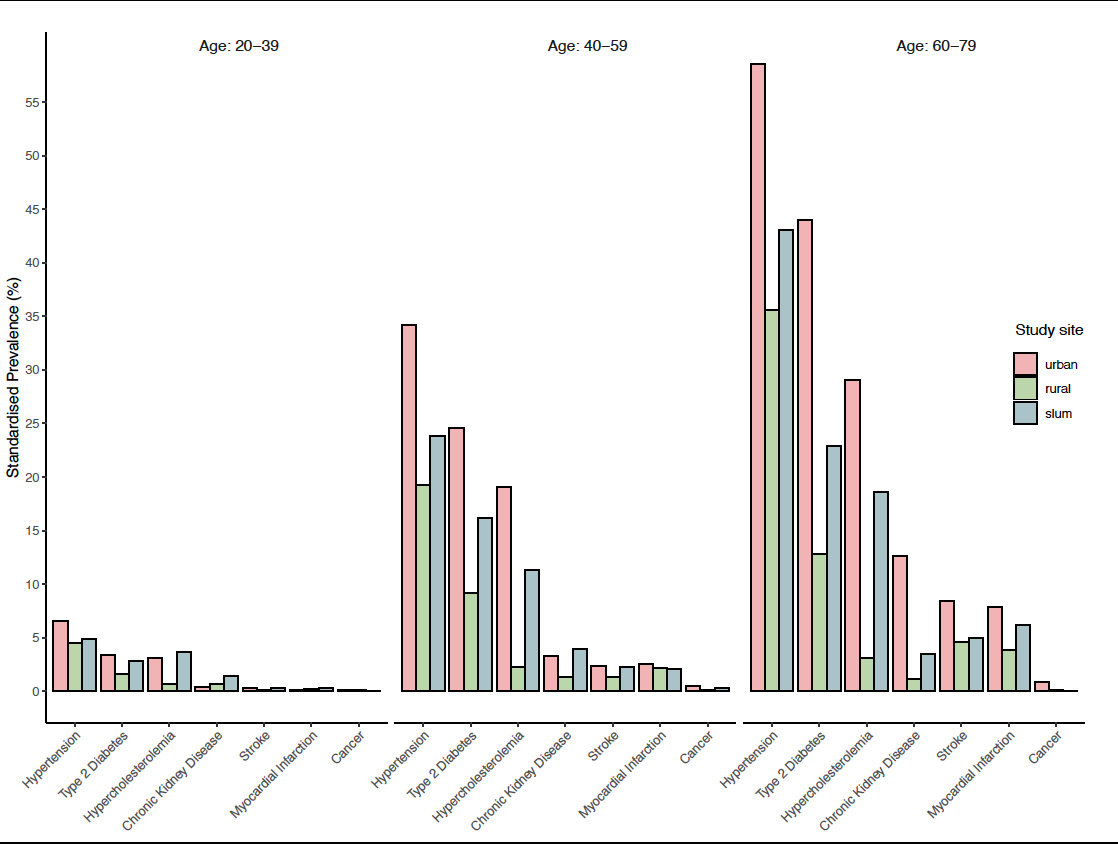


MI: myocardial infraction

Prevalence was standardised using the sex and age distribution of the Bangladesh 2022 population aged 20-79.

**Supplementary Figure 5.** Standardised prevalences of selected chronic conditions in participants aged 20-79, by sex and site

**
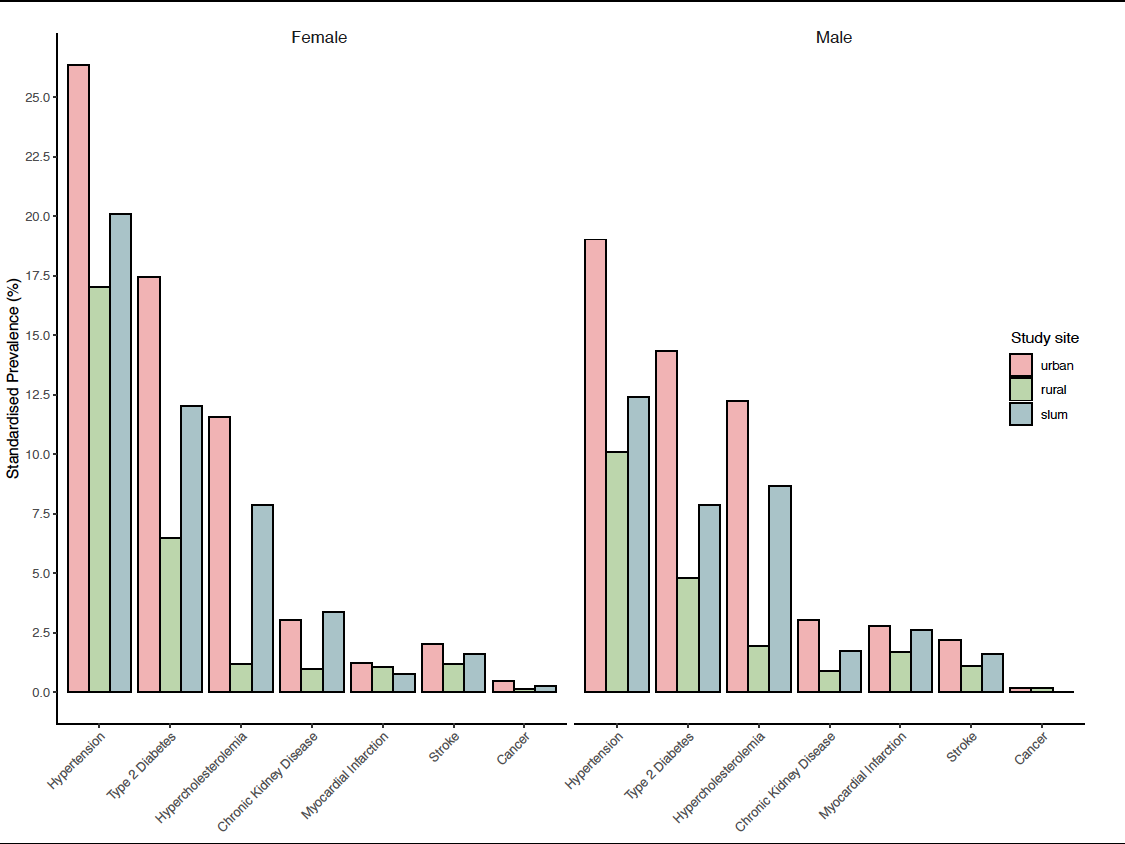
**

MI: myocardial infraction

Prevalence was standardised using the sex and age distribution of the Bangladesh 2022 population aged 20-79.

**Supplementary Figure 6.** Standardised prevalences of selected chronic conditions in participants aged 20-79, by sex, age and site


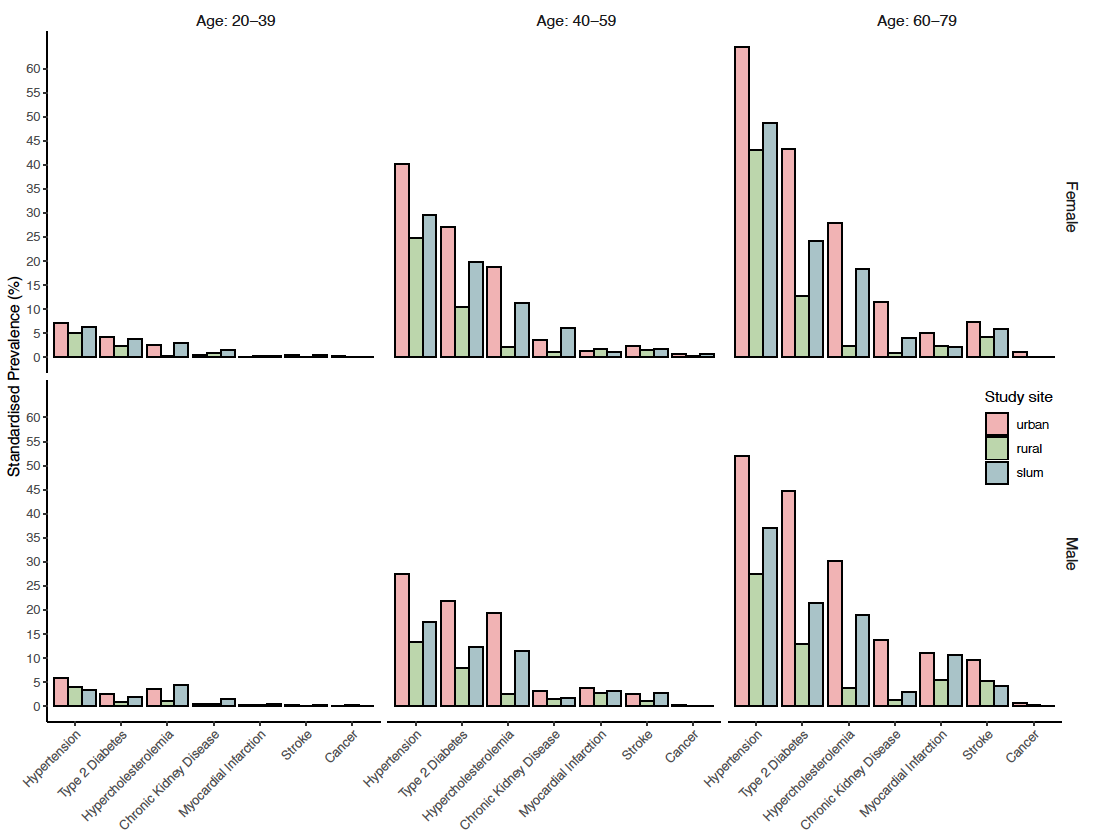


MI: myocardial infraction

Prevalence was standardised using the sex and age distribution of the Bangladesh 2022 population aged 20-79.
