# Supplementary material for: Cohort profile: the BangladEsh Longitudinal Investigation of Emerging Vascular and nonvascular Events (BELIEVE) cohort study
Source: BMJ Open. 2025 Jan 22;15(1):e088338. doi: 10.1136/bmjopen-2024-088338 (PMC11784369; doi:10.1136/bmjopen-2024-088338)
Supplement: Supplementary file 2 [file bmjopen-2024-088338.reviewer_comments.pdf]

PEER REVIEW HISTORY

BMJ Open publishes all reviews undertaken for accepted manuscripts. Reviewers are asked to complete a checklist review form and are provided with free text boxes to elaborate on their assessment. These free text comments are reproduced below.

ARTICLE DETAILS

Title (Provisional)

Cohort profile: The BangladEsh Longitudinal Investigation of Emerging Vascular and nonvascular Events (BELIEVE) cohort study

Authors

Chowdhury, Rajiv; Khan, Nusrat; Pennells, Lisa; Iurilli, Maria L C; Uddin Miah, Md Taslim; Monower, Md Mostafa; Rahman, K M Thouhidur; Samin, Sharraf; Saqeeb, Kazi Nazmus; Tasmin, Ishrat; Farrow, Eleanor; Farrow, Samantha; Michielsen, Ank; Perry, Catherine; Spackman, Sarah; van Coeverden, Charlotte; Walker, Matthew; Ahmed, Tahmeed; Ajioka, James; Awal, Khondker Abdul Abdul; Butterworth, Adam; Chatzidiakou, Lia; Feldmann, Jörg; Fenner, Richard; Flora, Meerjady Sabrina; Haque, Tuhin; Hawkes, Sarah; Islam, Syed Shariful; ISLAM, SIRAJUL; Jones, Roderic L; Kaptoge, Stephen; Khan, Kamrul Hasan; King, Lawrence; Luhar, Shammi; Malik, Abdul; Malik, Fazila-Tun-Nesa; Naved, Ruchira T; Naheed, Aliya; Popoola, Olalekan; Raqib, Rubhana; Shirin, Tahmina; Sutton, Stephen; van Daalen, Kim Robin; Wood, Angela; Griffin, Simon; Mascie Taylor, Nicholas; Khalequzzaman, Md.; Khan, Md Alfazal; Choudhury, Sohel Reza; Di Angelantonio, Emanuele; Danesh, John; The BELIEVE, study group*

VERSION 1 - REVIEW

**Reviewer**	**1**
**Name**	**Khodeir, Mostafa M**
**Affiliation**	**Cairo University, Pathology**
**Date**	**11-Aug-2024**
**COI**	**I declare that I have no competing interests in relation to this manuscript.**

---

1. Questionnaire development process:

- Provide a detailed description of the questionnaire development process.

- Explain how items were selected.

- State whether existing validated questionnaires have been adapted for this study.

2. Pre-testing and piloting:

- Describe the preliminary and pilot phases of the questionnaire.

- Include information on how the questionnaire was tested for clarity, relevance, and cultural appropriateness.

- Explain how feedback from these phases was incorporated into the final version.

3. Translation and back-translation:

- Detail the translation and back-translation processes if the questionnaire is translated into local languages to ensure the accuracy and cultural relevance of the translation process.

4. Validation:

- Discuss the validation studies conducted to ensure the reliability and validity of the questionnaire items.

5. Handling sensitive issues:

- Explain how sensitive questions (e.g., those related to health behaviors or personal medical history) were handled to ensure participant comfort and honest responses.

6. Quality control:

- Describe the quality control measures applied when administering the questionnaire, including details of how data entry errors were minimized and how consistency checks were conducted.

7. Additional documents:

- Provide the full questionnaire used.

8. Handling potential misclassification:

- Expand on the methods used to minimize misclassification of risk factors and health outcomes.

9. Handling missing data:

- Describe the strategies used to handle missing data in your analyses.

- Help readers understand how potential biases arising from missing data have been addressed.

10. Future directions:

- Discuss potential future research directions based on your findings.

- Highlight areas where more research is needed and how your study lays the groundwork for these future studies.

11- Supplementary Reporting: add details about supplementary reporting, as it will enhance the transparency and credibility of your study.

**Reviewer**	**2**
**Name**	**Ergün, Dilek**
**Affiliation**	**Selçuk Üniversitesi, , Faculty of Medicine,Department of Pulmonary Medicine**
**Date**	**13-Aug-2024**
**COI**	**I do not have any conflict of interest.**

The design of the article is well prepared. It will be useful in terms of accessing local data.

VERSION 1 - AUTHOR RESPONSE

Reviewer #1

1. Questionnaire development process: provide a detailed description of the questionnaire development process; explain how items were selected; state whether existing validated questionnaires have been adapted for this study.

DONE. We have now clarified on page 5, lines 154-155, that: "The study questionnaire was adapted to the Bangladesh context based on validated questionnaires used in previous large-scale studies."

2. Pre-testing and piloting: describe the preliminary and pilot phases of the questionnaire; include information on how the questionnaire was tested for clarity, relevance, and cultural appropriateness; explain how feedback from these phases was incorporated into the final version.

DONE. We have now clarified on page 5, lines 155-160, that: "The initial version of the structured questionnaire underwent preliminary testing with a small group of individuals from the target population to evaluate clarity, relevance, and cultural appropriateness. Following this, a larger group of individuals was involved for further validation and refinements, and adjustments were made to enhance readability and ensure logical flow, before finalising the questionnaire for the main study."

3. Translation and back-translation: detail the translation and back-translation processes if the questionnaire is translated into local languages to ensure the accuracy and cultural relevance of the translation process.

DONE. We have now clarified on page 5, lines 160-161, that: "To ensure both linguistic accuracy and cultural relevance, the questionnaire was translated into Bengali using a rigorous translation and back-translation process."

4. Validation: discuss the validation studies conducted to ensure the reliability and validity of the questionnaire items.

DONE. See responses #1 and #2 to Reviewer #1

5. Handling sensitive issues: explain how sensitive questions (e.g., those related to health behaviors or personal medical history) were handled to ensure participant comfort and honest responses.

DONE. We have now clarified on page 5, lines 170-172 that: "Direct computer entry by participants, rather than interviews, was employed to enhance privacy when answering sensitive questions, with the option to skip these questions if preferred."

6. Quality control: describe the quality control measures applied when administering the questionnaire, including details of how data entry errors were minimized and how consistency checks were conducted.

ALREADY DONE. on page 6, lines 203-207, it is stated that: "To enhance consistency in the collection of data, we trained staff extensively and adopted standardised approaches, validated instruments, and electronic data collection methods with built-in validity checks and queries. For example, the paper-free digital data collection platform involved extensive computerised checks to restrict missing values, duplications, inconsistencies, and outliers."

7. Additional documents: provide the full questionnaire used.

DONE. As clarified on page 5, line 169, "a copy of the study questionnaire is available in the Appendix."

8. Handling potential misclassification: Expand on the methods used to minimize misclassification of risk factors and health outcomes.

ALREADY DONE. On page 12, lines 428-433, it is stated that: "To help limit the effects for such potential misclassification, the BELIEVE study is supplementing self-reported data with use of objective measurements (e.g., assay of LDL-cholesterol, HbA1c, arsenic metabolites), inspection of health records kept by participants during household follow-up visits, use of previously validated "verbal autopsy" methods, and exploration of emerging potential linkages of study participants with digital health records kept at community healthcare and hospital levels."

9. Handling missing data: describe the strategies used to handle missing data in your analyses; help readers understand how potential biases arising from missing data have been addressed.

DONE. We have now clarified on page 9, line 297, that: "A complete case analysis was used to handle missing data". Furthermore, details of missing data are provided in the footnote of Table 2.

10. Future directions: discuss potential future research directions based on your findings; highlight areas where more research is needed and how your study lays the groundwork for these future studies.

DONE. We have now clarified in on page 11, lines 408-412, that: "The study has also collected a range of biological samples, including serum, plasma, whole blood, and nail samples stored in long-term repositories. Assay of these samples is enabling study of many molecular factors, laying foundations to advance understanding of disease pathways and potential therapeutic targets for treatment and prevention of NCDs in Bangladesh and beyond."

11. Supplementary Reporting: add details about supplementary reporting, as it will enhance the transparency and credibility of your study.

ALREADY DONE. Supplementary material provides details information and supplementary analyses.

-

Reviewer #2

No comments to address.
